# Exploring bismuth-doped polycrystalline ceramic Ba_0.75_Bi_0.25_Ni_0.7_Mn_0.3_O_3_: synthesis, structure, and electrical properties for advanced electronic applications

**DOI:** 10.1039/d3ra05038f

**Published:** 2023-08-10

**Authors:** Kais Iben Nassar, Faouzia Tayari, Majdi Benamara, Silvia Soreto Teixeira, Manuel Pedro F. Graça

**Affiliations:** a University of Sfax, Faculty of Sciences of Sfax, Materials Physics Laboratory B. P1171 3000 Sfax Tunisia; b University of Gabes, Faculty of Sciences of Gabes, Laboratory of Physics of Materials and Nanomaterials Applied to the Environment Erriadh 6079 Gabes Tunisia majdibenamara1@gmail.com; c I3N-Aveiro, Department of Physics, University of Aveiro 3810-193 Aveiro Portugal

## Abstract

This manuscript investigates the structural and electrical properties of a Ba_0.75_Bi_0.25_Ni_0.7_Mn_0.3_O_3_ (BNMO) perovskite compound synthesized through the sol–gel method. The orthorhombic crystal structure of the sample is confirmed by X-ray diffraction analysis. The electrical conductivity of BNMO is found to increase with frequency, indicating the presence of local charge carriers. The AC electrical conductivity follows Jonscher's equation, exhibiting a plateau at low frequencies and a power-law behavior at high frequencies. The activation energy for conduction is determined to be 0.654 eV. Impedance spectroscopy reveals the presence of grain and grain boundary contributions, which are modeled using an *R*–CPE combination circuit. Analysis of the electrical modulus demonstrates non-Debye type relaxation and indicates the presence of charge carrier hopping between Mn^2+^ and Mn^3+^ ions. The activation energy obtained from the relaxation peaks of the modulus is found to be 0.674 eV. The dielectric constant exhibits high values that increase with temperature. This observation suggests that the capacitance behavior holds promising potential for energy storage applications, making it a suitable candidate for various technological uses.

## Abstract 2

This manuscript investigates the structural and electrical properties of a Ba_0.75_Bi_0.25_Ni_0.7_Mn_0.3_O_3_ (BNMO) perovskite compound synthesized through the sol–gel method.

## Graphical abstract

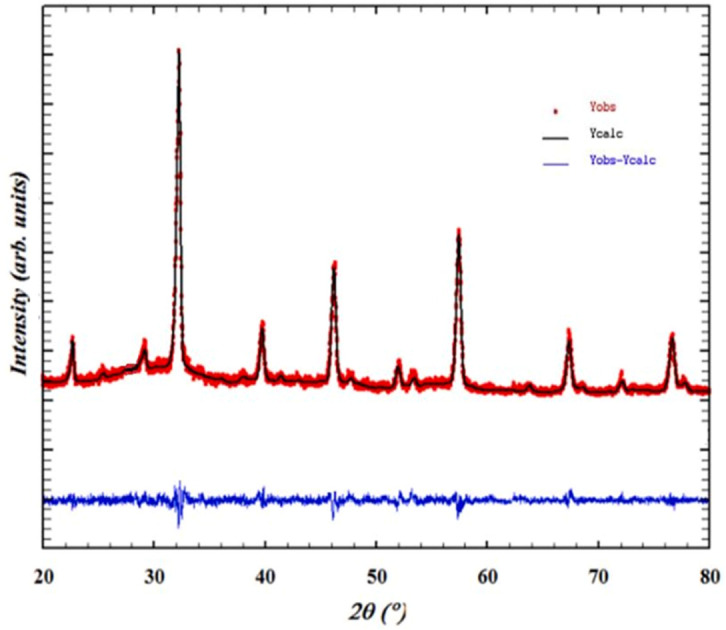

## 1. Introduction

Perovskite ferroelectric materials are highly intriguing due to their comparatively simple structure, which allows for the examination of theories and facile modification of physical properties through ion replacement.^1^ A new class of perovskite compounds, known as lead-free ferroelectric relaxers, has emerged as highly appealing, particularly for their high permeability and diffuse transitions, which are crucial for the capacitor dielectrics sector.^2^ These compounds share the characteristic of having two or more distinct cations occupying the same crystal site. Barium titanium (BaTiO_3_)-based ferroelectric materials have been the focus of several studies due to their exceptional dielectric, piezoelectric, pyroelectric, and electro-optical properties.^3^ However, the practical utilization of these materials relies on finding suitable substitutes that can maintain the relaxer phase at room temperature.^4^

Perovskite materials have garnered significant interest among scientists in recent years, owing to their wide range of physical features, including magnetism, electrical conductivity, and dielectric behavior.^5^ They find applications in various technological fields, such as telecommunications, energy storage technologies, magnetoresistive devices, microelectronic solar cells, ferromagnetic materials, and superconductors.^6^ These materials adhere to a basic framework, where the A-site is typically occupied by rare earth or alkaline earth ions, while the B′ and B′′ sites accommodate a variety of transition metals surrounded by oxygen anions, forming B′O_6_ and B′′O_6_ octahedra.^7^ Transition metal oxides, including perovskite materials, have received considerable attention in the realm of science and technology, and extensive investigations have been conducted.^8^ Notably, the LaMnO_3_ perovskite system, existing in a monoclinic or rhombohedral phase, has been a subject of particular focus.^9^

While previous research has explored the magnetic, electrical, and dispersive properties of perovskite materials, their technological applications differ from those of more conventional materials employed in solar cells, tunable electrical devices, gas and biosensors, among others.^10^ Furthermore, investigations have been conducted on the simulation of perovskite materials' rhombohedral phase to explore various challenges, including band structure, electronic state distribution, and absorption.^11^ Studies have also reported on the distorted dielectric characteristics and refractive index variations of perovskite materials caused by field frequency fluctuations. Moreover, these materials have been investigated for their potential as promising candidates for the next generation of electronics due to their dual eddy and magnetization properties.^12–14^ Additionally, perovskite oxides at room temperature can exhibit a variety of crystalline structures, such as cubic (*Pm*3), tetragonal (*I*4/*m*), monoclinic (*P*2_1_/*n*), and rhombohedral (*R*3*c*) systems.^15–17^ Among the double perovskite materials, the oxide BiBaFeZnO_6_, conforming to the general formula A′A′′B′B′′O_6_ (A′ = La, Bi, Sr, A′′ = Ba, B′ = Ni, B′′ = Nb, Mn, Fe), has been synthesized using the sol–gel method, with dielectric, electrical, temperature, and frequency dependencies being investigated.^18^ Furthermore, the complex electrical impedance analysis has provided insights into distinguishing true relaxation from non-Debye relaxation, supporting the concept that heat initiates the conduction process, as evidenced by the frequency dependence of electrical conductivity.^19^

The present work aims to examine the structural and electrical properties of a Ba_0.75_Bi_0.25_Ni_0.7_Mn_0.3_O_3_ (BNMO) sample synthesized using the sol–gel technology. Specifically, the electrical and modulus properties of the synthesized material, which vary with temperature and frequency, are investigated. By elucidating these properties, a deeper understanding of the characteristics of BNMO can be obtained, contributing to its potential applications, particularly in the field of next-generation electronics.

## 2. Experimental details

### 2.1. Synthesis

The Ba_0.75_Bi_0.25_Ni_0.7_Mn_0.3_O_3_ powder was synthesized using a sol–gel process with Ba(NO_3_)_2_, Bi(NO_3_)_3_, Ni(NO_3_)_2_, and Mn(NO_3_)_2_ as precursor nitrates. Distilled water was used to dissolve the metal nitrates at the stoichiometric concentration, forming a solution. The solution underwent hydrolysis and polycondensation reactions, resulting in the formation of a gel like precursor. The gel was then dried to obtain a solid powder.

To prepare pellets of Ba_0.75_Bi_0.25_Ni_0.7_Mn_0.3_O_3_ (BNMO), the synthesized powder was compressed into pellets with dimensions of 8.4 mm in diameter and 1.5 mm in thickness. This pelletization step ensured uniformity and facilitated subsequent characterization and measurement procedures. Heat treatment was applied to enhance the structural and chemical stability of the BNMO material. The pellets were subjected to heat treatment at different temperatures, specifically 600 °C, 700 °C, and 950 °C, for 24 hours. This thermal treatment promoted crystallization and eliminated any residual organic components from the sol–gel process, resulting in the desired BNMO phase.

### 2.2. Characterizations

The crystalline phase of the powder was confirmed using X-ray diffraction (XRD) analysis performed on a Bruker D8 diffractometer with a copper anticathode (*λ* CuKα = 1.5406 Å) operating at 40 kV and 40 mA. The XRD measurements covered a 2*θ* range from 5° to 80° with a step size of 0.02°, providing detailed information about the crystal structure and phase purity of the sample. Scanning Electron Microscopy (SEM) equipped with Energy Dispersive X-ray Spectroscopy (EDS) was utilized for the analysis of particle microstructure and elemental composition. The SEM analysis was carried out using a FEI Quanta 650 FEG scanning electron microscope operating at 15 kV. The EDS system enabled the identification and mapping of elements present in the sample, providing insights into the chemical composition and spatial distribution of different elements.

To facilitate electrical measurements, the pelletized sample was coated with a thin layer of silvery oxide on both sides to ensure ohmic contact. This contact layer was crucial for accurate electrical characterization. Electrical measurements were performed using an Agilent 4294 A analyzer, applying an AC signal with an amplitude of 50 mV over a frequency range spanning from 100 Hz to 1 MHz. This allowed for the investigation of electrical properties, such as conductivity and impedance, as a function of frequency.

## 3. Results and discussion

### 3.1. Structural properties

**Fig. 1:**
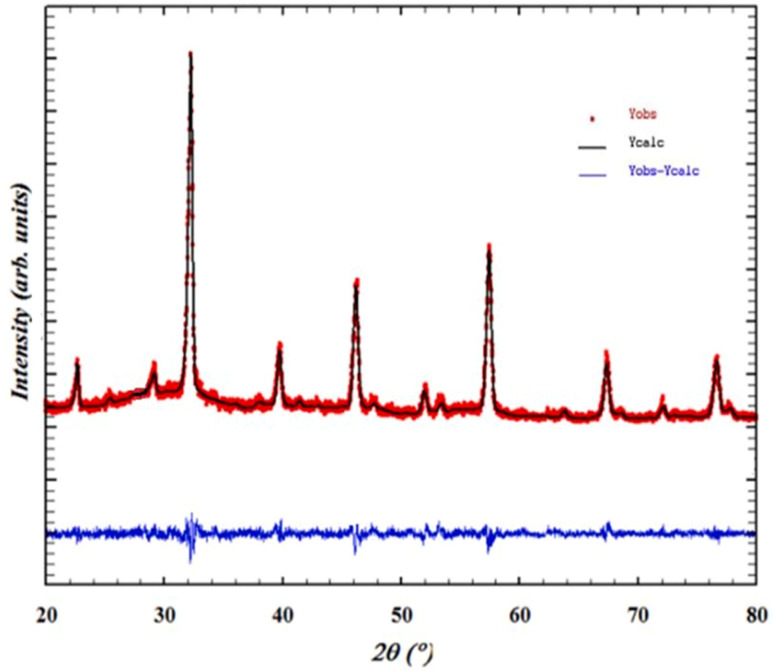
XRD patterns refined by FULLPROOF software of the prepared BNMO sample.

To assess the purity of our chemical compound, we conducted structural and morphological studies using various analytical techniques. The room temperature diffraction pattern of our BNMO sample was obtained using a powder X-ray diffractometer, as shown in Fig. 1. The diffraction pattern revealed that the structure of the compound is orthorhombic, belonging to the *Pnma* space group. The refinement of the structure was performed using the FULLPROOF tool,^20,21^ confirming the successful crystallization of our molecule, which exhibited distinct large peaks.

The Rietveld method was employed to refine the structure further, yielding important parameters related to the unit cell (*a*, *b*, and *c*), and the volume of the unit cell (*V*). These refinement results are summarized in Table 1, which includes the structure factor (*R*_f_), Bragg factor (*R*_p_), and fit goodness (*χ*^2^). Additionally, the distortion of the ideal perovskite structure was investigated by calculating the Goldschmidt tolerance factor using the following formula:^22^1
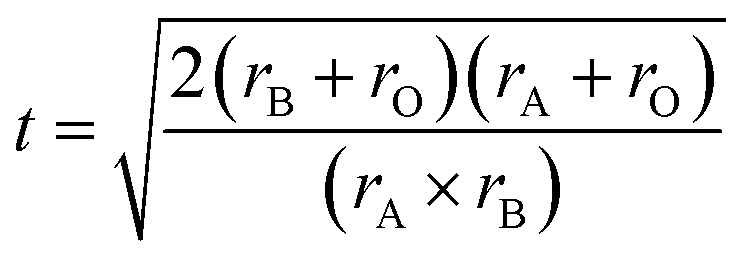
where *r*_A_ corresponds to the ionic radius of the cations at sites A, *r*_B_ corresponds to the ionic radius of the cations at sites B, and *r*_O_ represents the ionic radius of oxygen. For our sample, the ionic radii were determined based on Shannon's values: *r*(Bi^3+^) = 0.96 Å, *r*(Ba^2+^) = 1.35 Å, *r*(Ni^2+^) = 0.69 Å, *r*(Mn^4+^) = 0.64 Å, and *r*(O^2−^) = 1.4 Å.^23,24^ The calculated tolerance factor *t* for our sample was found to be 0.954, confirming the orthorhombic crystal system of the material. Moreover, the Scherrer formula was employed to determine the crystallite size using the equation:^25^2
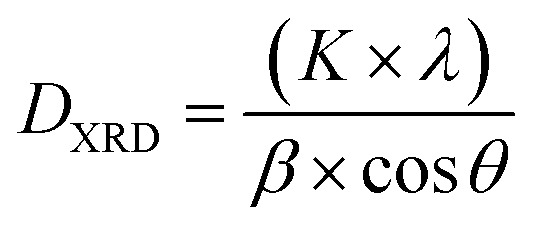
in this equation, *K* represents the form factor (*K* = 0.9), *λ* is the wavelength of the X-ray used (*λ* = 1.5406 Å), *β* is the FWHM-corrected peak width, and *θ* is the diffraction angle of the most intense peak. The term *β*^2^ is defined as *β*_m_^2^ − *β*_s_^2^, where *β*_m_ corresponds to the full width at half maximum (FWHM) of the experimental sample, and *β*_s_ represents the FWHM of a typical silicon sample.^26^ Due to the nanoscale crystallization of our product, the Scherrer formula determined an average crystallite size of approximately 35 nm, supporting the suitability of the sol–gel method employed for its preparation.

**Table 1:** Refined structural parameters of Ba_0.75_Bi_0.25_Ni_0.7_Mn_0.3_O_3_ compound at room temperature

Paramaters	*a* (Å)	*b* (Å)	*c* (Å)	*V* (Å^3^)	*χ* ^2^	*R*_f_	*R*_B_
Values	5.539	7.945	7.686	243.229	1.69	1.56	1.76

### 3.2. Morphological studies

**Fig. 2:**
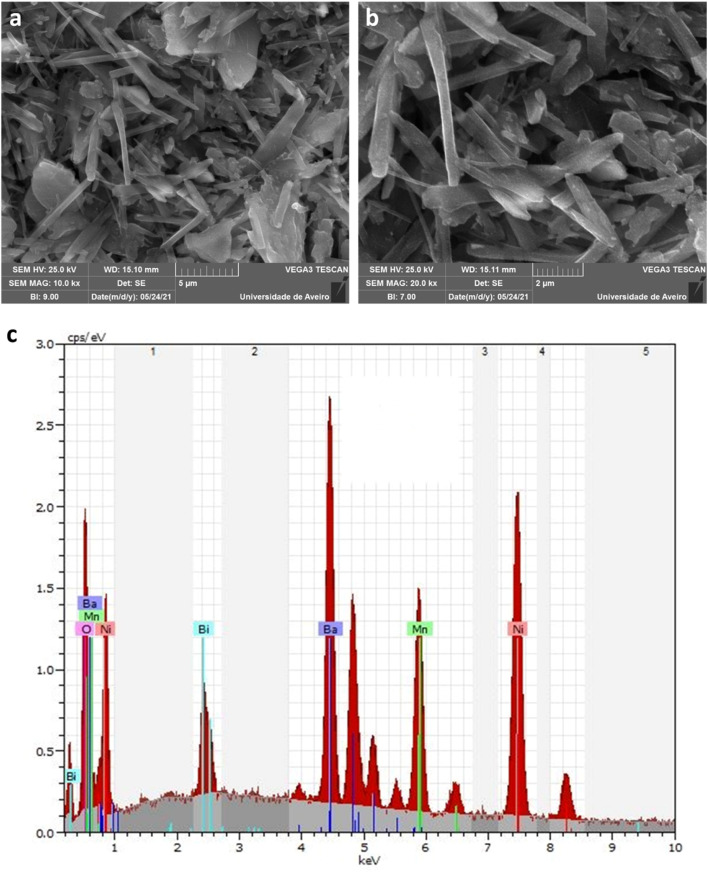
SEM micrograph with (a) 5 μm and (b) 2 μm as scales. (c) EDS spectrum of BNMO compound.

For the investigation of morphological and chemical composition, we utilized a scanning electron microscope (SEM) to analyze the BNMO sample, which was prepared using the sol–gel technique. Fig. 2a displays an SEM image of the material at a 5 μm scale, revealing a significant presence of micrometric granulometry with diverse and non-homogeneous shapes. Our structure exhibits a unique combination of nanosheets and nanowires, with some of the nanowires reaching lengths of approximately 6.8 μm. To accurately estimate the grain sizes, we further analyzed the SEM image at a 2 μm scale (Fig. 2b) using the “Image-J” program. The dimensions of the nanowires were found to have lengths ranging from 3.1 to 6.8 μm and widths between 0.5 and 0.7 μm. The presence of these nanowires in our structure holds the potential to significantly enhance the material's electrical conductivity. Moreover, Fig. 2c presents the energy-dispersive X-ray spectroscopy (EDS) spectrum of the BNMO sample, with distinct peaks representing the constituent elements of the material, namely Ba, Bi, Ni, Mn, and O. The EDS analysis confirms the successful incorporation of these elements into the BNMO structure.

### 3.3. Measurement of electrical conductivity

**Fig. 3:**
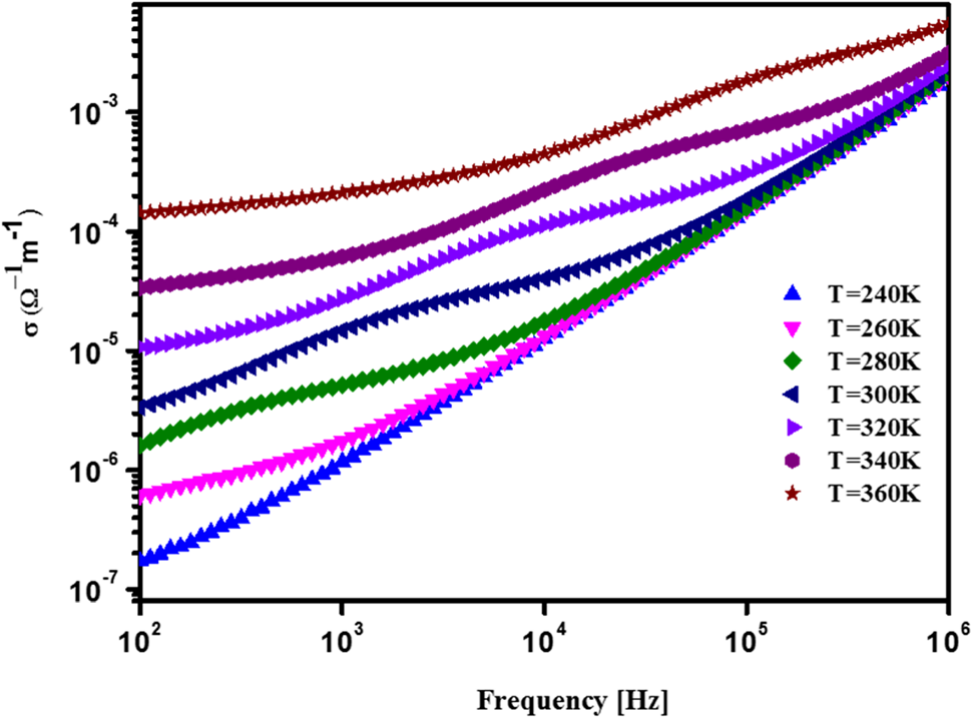
Conductivity spectra over a temperature range of BNMO.

The measurement of electrical conductivity is essential for understanding the conduction mechanism in magnetite materials. In this study, we conducted experimental conductance measurements and impedance spectroscopy readings to determine the electrical conductivity of the material. Fig. 3 illustrates the AC electrical conductivity of BNMO as a function of frequency and temperature, revealing intriguing insights into its behavior.

The results demonstrate that the conductivity increases at higher frequencies, indicating the involvement of local charge carriers in the conduction process. The figure exhibits two distinct regions, each offering valuable information about the conduction mechanism.

At lower frequencies, a constant DC conductivity is observed, as indicated by the plateau in the graph when plotted against the angular frequency (*ω* = 2*f*). This plateau suggests that the conduction is governed by the long-distance translational motion of charge carriers. Furthermore, the DC conductivity value of the sample shows an upward trend with increasing temperature, implying its semiconducting nature. To characterize the conductivity dispersion at higher frequencies, we employ Jonscher's equation:^27^

3 *σ*_ac_(*ω*) = *σ*_dc_ + *Aω*^s^

Here, *A* represents a temperature-dependent constant, *σ*_dc_ is the direct current conductivity, *s* (0 < *s* < 1) is a dimensionless variable that characterizes the dispersion in the material, and *ω* denotes the angular frequency. The change in the exponent *s* reflects variations in the material's polarizability, which arises due to energy barrier transitions between successive charge carrier sites.^28^ This unique characteristic allows glasses, amorphous semiconductors, ionic conductors, and certain crystals to conduct electricity.^29^

This study on BNMO material's electrical conductivity offers crucial advantages from the existing data. Through experimental conductance measurements and impedance spectroscopy readings, the material's behavior was comprehensively analyzed as a function of frequency and temperature. The results revealed the involvement of local charge carriers at higher frequencies and a constant DC conductivity at lower frequencies, indicative of long-distance translational motion of charge carriers and its semiconducting nature, respectively. Additionally, employing Jonscher's equation to characterize conductivity dispersion at higher frequencies enabled quantification of polarizability and energy barrier transitions between charge carrier sites. These valuable insights have practical implications in designing electronic devices operating at specific frequencies, developing temperature-sensitive components, and tailoring materials for diverse electrical and electronic applications, ranging from electronics to energy systems, thus advancing the understanding and potential utilization of magnetite materials.

**Fig. 4:**
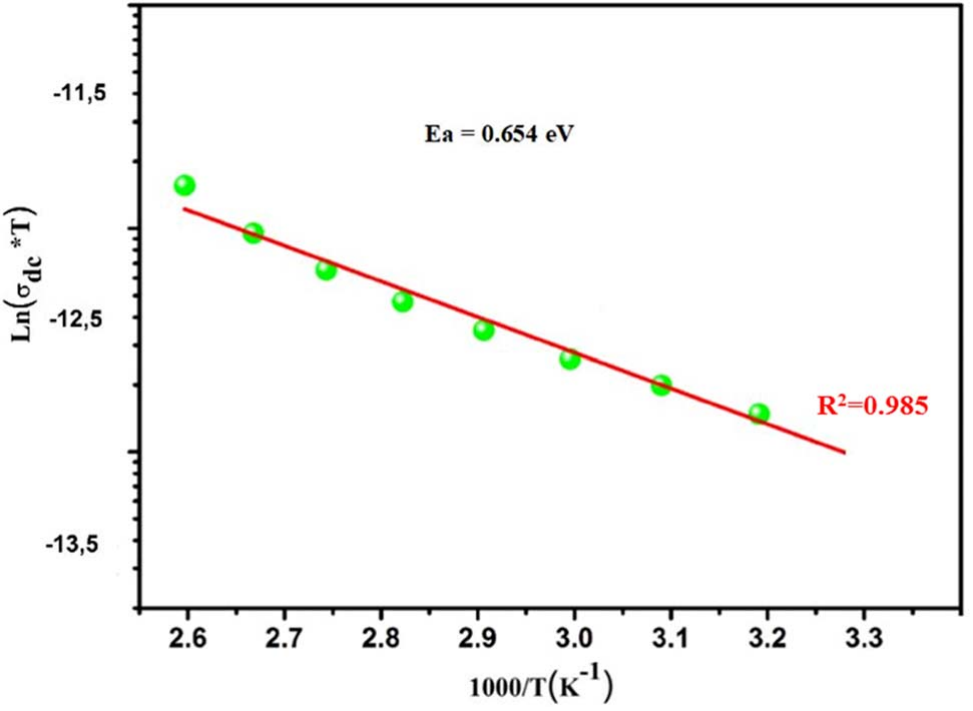
Temperature dependence of ln(*σ*_dc_ × *T*) *vs.* 1000/*T* plots for the BNMO sample.

The data in Fig. 4 were fitted using eqn (3). To determine the activation energies associated with this conduction mechanism, we apply Arrhenius' law. Fig. 4 presents the fluctuations in ln(*σ*_dc_ × *T*) plotted against 1000/*T*, enabling the calculation of activation energies. The equation utilized for analysis is as follows:4
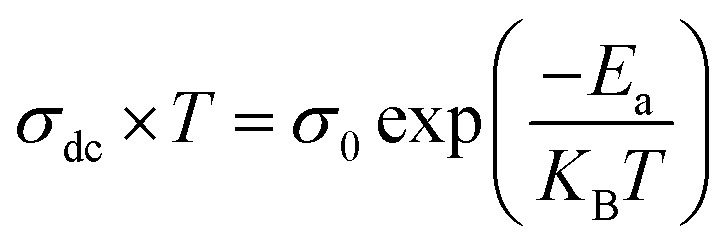
in this equation, *K*_B_ represents the Boltzmann constant, *σ*_0_ is the pre-exponential term, and *E*_a_ corresponds to the activation energy. The graph demonstrates a linear relationship at high temperatures, providing evidence that the conduction mechanism in this material is thermally driven. By examining the slope at high temperatures, we can determine the activation energies. The data clearly show that an increase in temperature leads to higher conductivity, as observed from the linearity in our sample. Notably, the BNMO molecule exhibits an activation energy of 0.654 eV. The goodness of fit, represented by *R*^2^, was 0.985, indicating an excellent fit for our data.

### 3.4. Impedance analysis

**Fig. 5:**
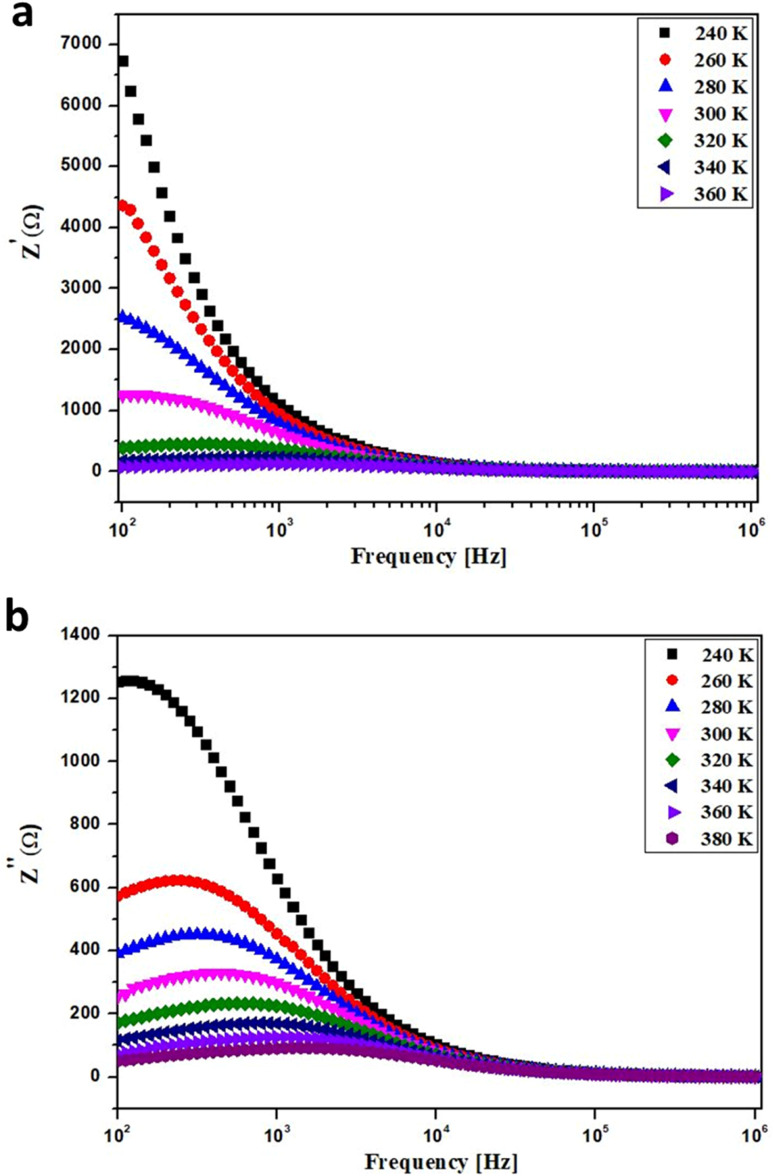
Frequency dependence of (a) *Z*′ and (b) *Z*′′ for BNMO sample.

Impedance spectroscopy was employed to investigate the complete electrical behavior of the system, including resistive and conductive properties, dielectric relaxation processes, and polycrystalline samples. From the experimental data, we calculated the imaginary (*Z*′′) and real (*Z*′) impedance components. These components are related as follows:^30^5
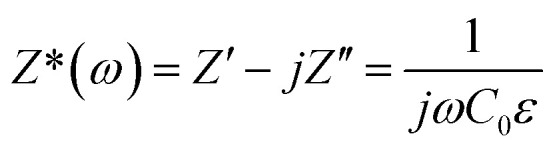
in the temperature range of 240–380 K, Fig. 5a illustrates the variation of the real impedance component (*Z*′) with frequency for BNMO nanoparticles. As observed, *Z*′ tends to increase at low frequencies before decreasing with increasing frequency. In the high-frequency zone, *Z*′ remains constant due to the discharge of space charges. When temperatures rise, the barrier characteristics of compounds diminish. The observed variation in *Z*′ for our samples is consistent with previous studies reported in the literature.^31^

Fig. 5b depicts the variation of the imaginary impedance component (*Z*′′) as a function of frequency. The highest *Z*′′ value is observed in the low-frequency range, which shifts to higher frequencies as temperature increases. Additionally, typical temperature-related peak broadening is observed. The behavior of *Z*′′ can be attributed to the presence of immobile species at low temperatures and specific vacancies at high temperatures. The behavior of *Z*′′ in the high-frequency range can be attributed to the accumulation of space charge in our sample. These findings align with previous studies in the literature.^32^

### 3.5. Nyquist analysis

**Fig. 6:**
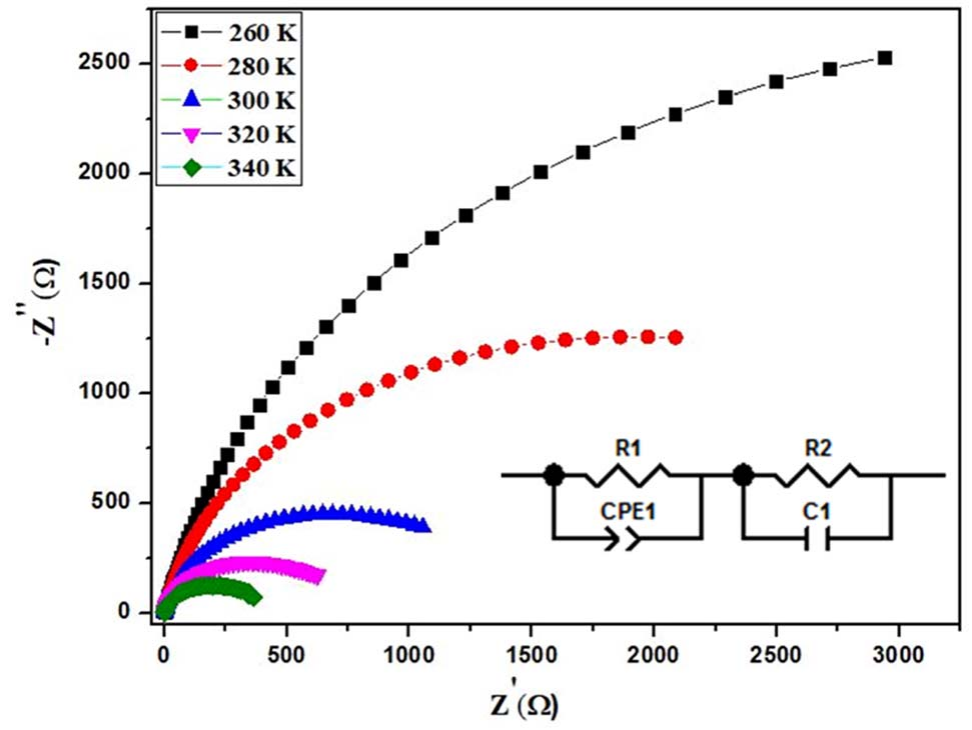
Nyquist diagrams.

To analyze the contributions of grains and grain boundaries to the transport process in our compounds, Nyquist modeling was performed. Fig. 6 presents the equivalent circuit of our samples obtained using Z view software, along with the simulated *Z*′′ curves as a function of *Z*′. The use of Z-view software facilitated impedance measurements. The sample device consists of two series-connected circuits combining resistance (*R*) and constant phase element (CPE), representing the contributions of grain boundaries (*R*_gb_, *C*_gb_) and grains (*R*_g_, *C*_g_), respectively. The impedance of the constant phase element (CPE) is described by the following relation:^33^6
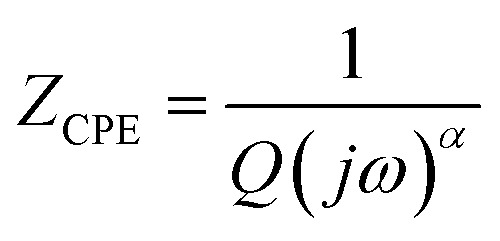
Here, *Q* is a proportional factor, *w* is the angular frequency, and *α* is an empirical exponent close to 1. The behavior of the CPE can be compared to commonly used components in similar circuits, depending on the value of *α*. When *α* is close to zero, the CPE reduces to a resistance, while *α* = 1 corresponds to an ideal capacitive element. The derived parameters were obtained through fitting and are presented in Table 2. The good agreement between the fitted results and experimental data is evident.^34^

**Table 2:** Equivalent circuit parameters values for BNMO sample

Temperature (K)	240	260	280	300	320	340	360	380
*R*_1_ (×10^4^ Ω)	21.67	18.84	15.21	12.53	10.34	9.26	7.38	6.67
CPE_1_ (×10^−11^ F)	5.97	4.41	3.15	2.18	2.67	2.42	6.23	2.77
*α*	0.747	0.829	0.984	0.955	0.941	0.921	0.936	0.856
*R*_2_ (×10^3^ Ω)	7.57	6.51	5.64	5.33	4.19	4.64	4.60	3.57
*C*_1_ (×10^−11^ F)	13.92	12.73	11.42	9.23	8.74	7.45	6.32	5.94

### 3.6. Modulus analysis

To understand the characteristics of grains and grain boundaries at different frequencies, we need to explore the complex electrical modulus. The equation below was used to calculate the real and imaginary parts of the electric modulus using impedance:

7 M* = *M*′ + *jM*′′ = *jωC*_0_Z*

**Fig. 7:**
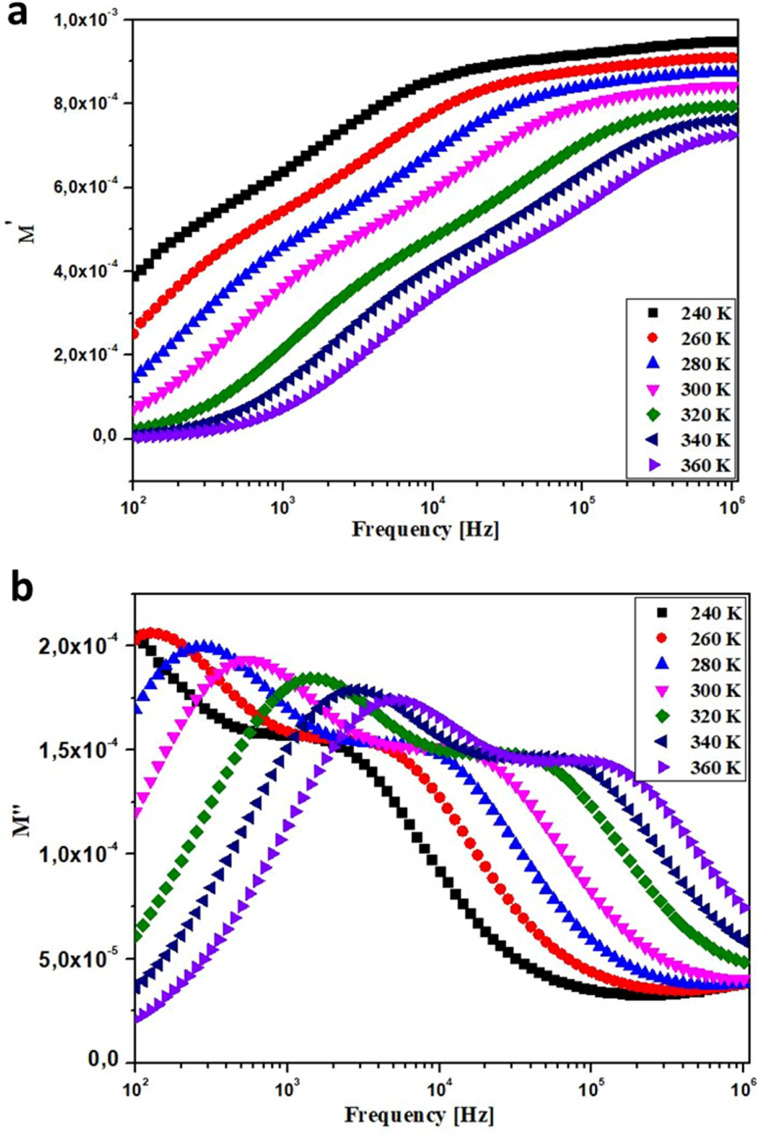
Frequency dependence of (a) *M*′ and (b) *M*′′ at various temperature for BNMO sample.

Here, *C*_0_ represents the vacuum capacitance of the cell. The electric modulus *M*′ remains constant and approaches zero at low frequencies, as shown in the plots of *M*′ at different temperatures in Fig. 7a. This indicates that electrical polarization has minimal effects. The curves increase linearly with frequency, indicating that conduction occurs through charge carriers hopping between Mn^2+^ and Mn^3+^ ions over short distances.^33^Fig. 7b shows the variation of the imaginary component *M*′′ with an increasing element as frequency rises. At each temperature, an asymmetric peak is observed, from which the maximum conduction loss can be calculated. Furthermore, as temperature increases, the asymmetric peak shifts to higher frequencies. This can be explained by the acceleration of the relaxation process due to the heat activation of mobile ions. The behavior of the modulus can be compared to various literary references.^35^ This phenomenon can be understood by considering a mountain that separates two geographically distinct regions. In the low-frequency domain, the hopping conduction process enables mobile ions to move over long distances. However, in the high-frequency region, the mobile ions are restricted and become immobile due to potential wells.^35^

**Fig. 8:**
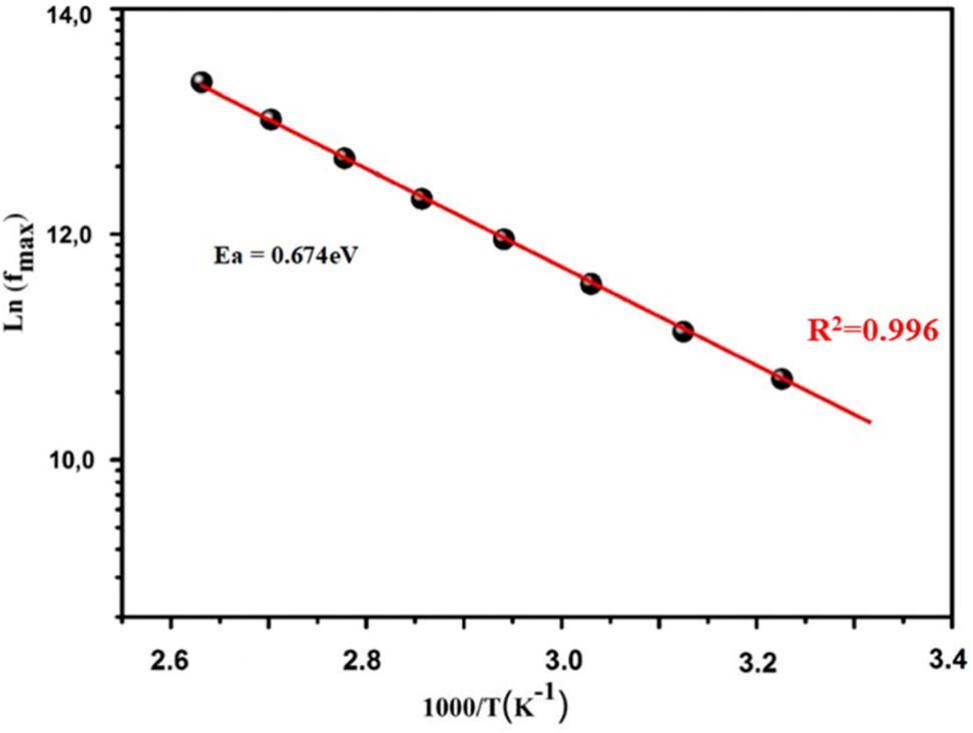
Arrhenius plots show dependence *f*_max_ as a function of 1000/*T*.

The maximum value of *M*′′ is denoted as 
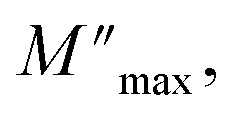
 and its corresponding frequency is *f*_max_ in the *M*′′(*f*) graphs presented here. From Fig. 7b, the relaxation frequencies *f*_max_ are determined, and ln(*f*_max_) is plotted as a function of 1000/*T*. This relationship can be described by the Arrhenius plot,^36^ given as:8
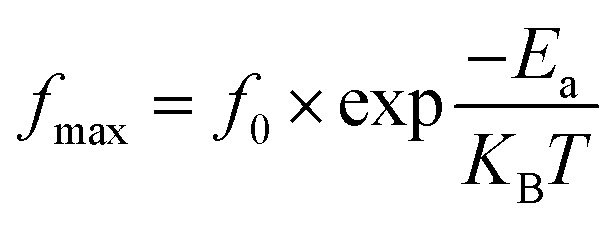
Here, *T* represents the temperature, *E*_a_ is the activation energy, and *K*_B_ is the Boltzmann constant. *f*_0_ is the pre-exponential factor. Fig. 8 shows that the activation energy is measured to be 0.674 eV. The similarity in charge carriers involved in both the relaxation process and the conduction mechanism is supported by the close relationship between the activation energy derived from conductivity and the frequency corresponding to the relaxation peaks of the imaginary part of the modulus (*M*′′). The obtained goodness of fit (*R*^2^) value of 0.996 demonstrates a strong fit for our data.

### 3.7. Dielectric properties

**Fig. 9:**
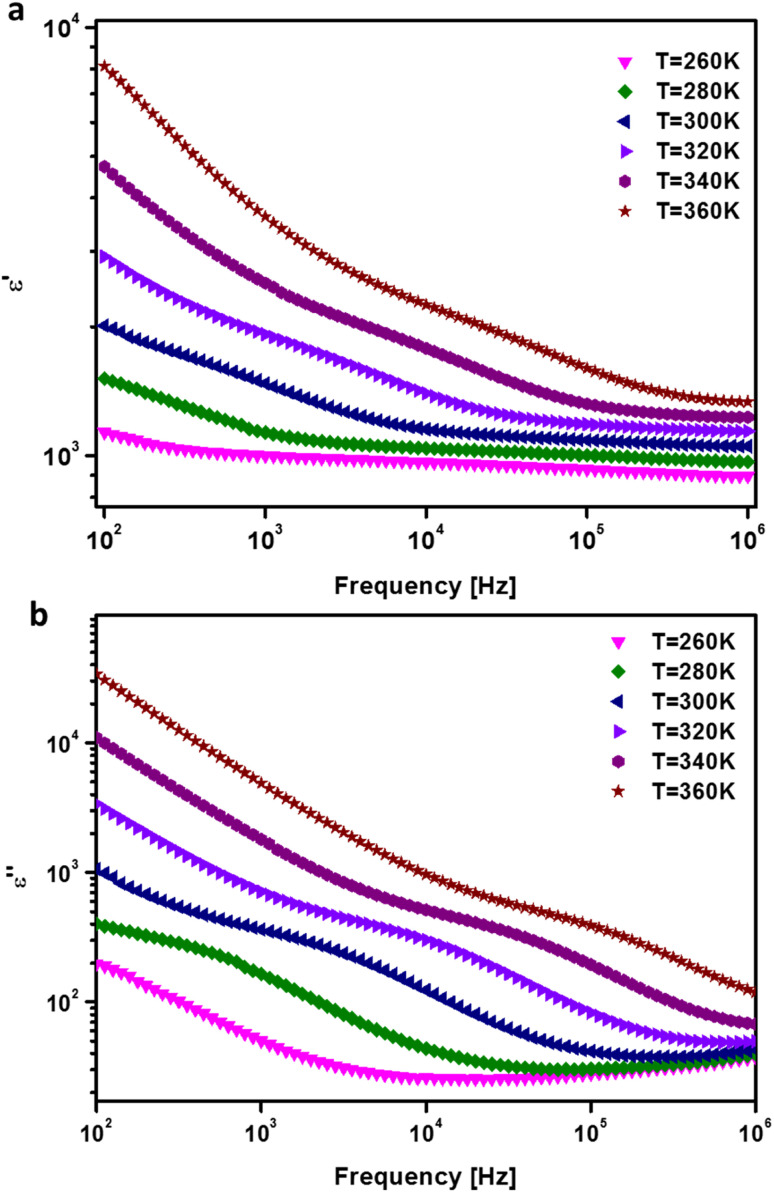
Frequency dependences of (a) the real and (b) the imaginary parts of the electrical permittivity of the prepared BNMO sample.

The dielectric constant's variation with frequency can be attributed to dispersion caused by the Maxwell–Wagner interfacial polarization, in accordance with Koop's phenomenological theory.^37^ The complex relative dielectric permittivity, expressed as *ε* = *ε*′ + i*ε*′′, encompasses the real (*ε*′) and imaginary (*ε*′′) components of the permittivity. Fig. 9a illustrates the evolution of *ε*′ at different temperatures for the prepared compound. Notably, at low frequencies, *ε*′ exhibits higher values due to the double-exchange interaction between the ferromagnetic coupled Mn^3+^ and Mn^2+^ ions, leading to enhanced energy storage. Consequently, with increasing temperature, the charge accumulation at the grain boundary intensifies. Furthermore, an increase in *ε*′ is observed with rising temperature, demonstrating greater stability. Typically, the evolution of *ε*′ is attributed to four types of polarizations, namely, interfacial, dipolar, atomic, and electronic. Specifically, at low frequencies, the temperature-dependent evolution of *ε*′ is correlated with dipolar and interfacial polarizations.^38^

The frequency and temperature dependence of the imaginary part of permittivity (*ε*′′) for the BNMO compound is presented in Fig. 9b. It is evident from the figure that *ε*′′ decreases as frequency increases. Generally, the imaginary part of permittivity is influenced by the conduction mechanism and polarization.^39^ At lower frequency regions, the exchange of electrons between the ferromagnetic coupled Mn^3+^ and Mn^2+^ ions occur in response to the alternating electric field, leading to high conductivity. Conversely, at higher frequency regions, dipoles respond to the alternating field, resulting in a reduced dielectric constant. Additionally, the absence of a relaxation peak for all temperatures confirms the non-Debye behavior.^40^

## 4. Conclusions

In this study, the BNMO perovskite was synthesized using the traditional sol–gel method. The X-ray diffraction analysis confirmed the formation of an orthorhombic structure with *Pnma* group space. Electrical and dielectric measurements were performed using complex impedance spectroscopy at frequencies ranging from 100 Hz to 10^6^ Hz and temperatures ranging from 260 K to 380 K. The observed behavior of *Z*′′ and *M*′′ reaching their maximum values at different relaxation frequencies suggests the presence of a charge carrier that is in close proximity. The modulus analysis of the sample indicated the presence of non-Debye type relaxation, highlighting the complex nature of the dynamic process. Interestingly, the normalized scaling behavior demonstrated that the dynamic process was independent of temperature. This independence implies that the conductivity of the investigated compound is not strongly influenced by temperature variations. Frequency-dependent dielectric variation is attributed to interfacial polarization and enhanced energy storage at low frequencies due to Mn^3+^ and Mn^2+^ ions' double-exchange interaction. *ε*′ increases with temperature, associated with dipolar and interfacial polarizations. BNMO's *ε*′′ shows a decreasing trend with frequency and temperature. High conductivity at lower frequencies results from electron exchange between Mn^3+^ and Mn^2+^ ions, while higher frequencies reduce the dielectric constant, confirming non-Debye behavior. Overall, the results of this study provide valuable insights into the structural and electrical properties of the BNMO perovskite synthesized through the sol–gel method. The understanding gained from this research can contribute to the further exploration and optimization of the material for potential applications in various electronic devices and systems.

## Conflicts of interest

The authors affirm that they did not accept any money, grants, or other assistance for the creation of this manuscript.

## Supplementary Material
